# Temporal-order information can be maintained in non-conscious working memory

**DOI:** 10.1038/s41598-019-42942-z

**Published:** 2019-04-24

**Authors:** Darinka Trübutschek, Sébastien Marti, Stanislas Dehaene

**Affiliations:** 1 Ecole des Neurosciences de Paris Ile-de-France, 15 rue de l’école de médecine, 75006 Paris, France; 2 Sorbonne Université, UPMC Université Paris 06, 4 place Jussieu, 75005 Paris, France; 3 Cognitive Neuroimaging Unit, CEA DSV/I2BM, INSERM, Université Paris-Sud, Université Paris-Saclay, NeuroSpin centre, 91191 Gif/Yvette, France; 4 Collège de France, 11 Place Marcelin Berthelot, 75005 Paris, France

**Keywords:** Human behaviour, Working memory, Consciousness

## Abstract

Classical theories hold conscious perception and working memory to be tightly interwoven. Recent work has challenged this assumption, demonstrating that information may be stored for several seconds without any subjective awareness. Does such non-conscious working memory possess the same functional properties as conscious working memory? Here, we probe whether non-conscious working memory can maintain multiple items and their temporal order. In a visual masking task with a delayed response, 38 participants were asked to retain the location and order of presentation of two sequentially flashed spatial positions, and retrieve both after a 2.5 second delay. Even when subjective visibility was nil, subjects’ objective forced-choice performance exceeded chance level and, crucially, distinct retrieval of the first and second location was observed on both conscious and non-conscious trials. Non-conscious working memory may therefore store two items in proper temporal order. These findings can be explained by recent models of activity-silent working memory.

## Introduction

Until recently, conscious perception and working memory were thought to be closely related, both enabling the short-term maintenance of information^1–3^ and relying on elevated, sustained neural activity^4–7^. Empirical evidence has since challenged these prevailing views. Masked items, that participants decline to have seen consciously, may be encoded into and maintained in working memory^8–10^, and working memory itself may operate without subjective awareness^11,12^. Using magnetoencephalography, we have recently shown the existence of a genuine form of non-conscious working memory storing information in “activity-silent” brain states^13^, presumably via short-term synaptic plasticity^14^. However, such non-conscious working memory is a recent discovery, and we still know very little about its functional properties. Is it a fully-fledged working memory system, with characteristics similar to the ones of conscious working memory? Or should it be conceived of as a restricted special-purpose system, with limited functionality?

Here, we probe one of the most defining features of conscious working memory: the ability to store multiple items in correct temporal order. Historically, research on conscious working memory has focused on the short-term maintenance of ordered information, including lists of digits, letters, or words^15–18^. Most contemporary conceptualizations of working memory have evolved from these early findings. Any working memory system in the conscious sense should thus be able to accommodate the storage of multiple items in addition to their temporal order. However, whether this is also the case for non-conscious working memory is currently not clear.

Research on non-conscious working memory has almost exclusively investigated the storage of a single sensory item. Participants either had to remember the orientation of a Gabor patch^10,11^, a spatial location^13^, or a number/letter^8^. To our knowledge, there have only been two studies to date, in which there were either two simultaneously presented memory stimuli^10^, or the initial memorandum consisted of two simultaneously presented feature dimensions (i.e., object identity and its spatial location^9^). However, sample size was extremely small (i.e., *N* = 9) and the delay period fairly short (i.e., 2 s) in the first experiment, and, in the latter case, automatic object-based attention may have integrated both features into a single object file, thus reducing the remembered stimulus to a single, bound representation^19^. There is thus very little, if any, empirical evidence that would be able to speak directly to the storage of multiple non-conscious items and no such information at all when considering temporal order.

We here set out to address these key unknowns by confronting non-conscious working memory with multiple, independents items and their temporal order. Specifically, we aimed to determine (1) whether more than a single item may be maintained simultaneously in non-conscious working memory, and (2) if non-conscious storage includes order information. We were able to answer both of these questions in the affirmative: Two subjectively unseen target stimuli as well as their order could be retained for several seconds. As such, these results critically expand the realm of non-conscious working memory, further challenging predominant models of the nature of working memory.

## Results

**Figure 1:**
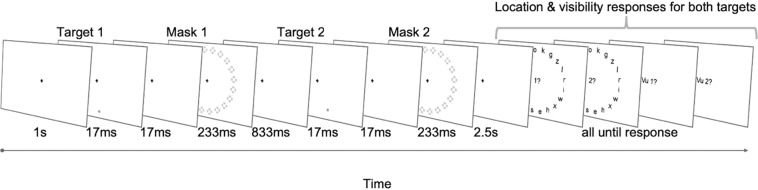
Experimental design. Two individually masked target squares were flashed in 1 out of 20 positions. Each possible combination of angular distance between the two targets occurred once during the course of the experiment, such that, on a small subset of trials (i.e., 5%), successive targets appeared in the exact same spatial location. On 20% of trials, the presentation of either one or both targets was omitted and replaced by the display of a blank screen (target-absent control). Participants were instructed to perform two consecutive tasks after a long delay: First, they had to localize both targets in the order they had appeared. Then, they were to rate their visibility for each target on a 4-point scale. Critically, subjects had to complete both tasks, even when they had not seen the squares. In that case, they simply were to guess a position.

Participants completed a modified version of a spatial delayed-response paradigm, requiring the short-term maintenance of two sequentially presented spatial locations and their order of appearance (Fig. 1). Each target was flashed in 1 of 20 possible positions, selected independently of each other with replacement, and immediately masked. After a 2.5 s delay period, subjects first localized both targets (by typing the random letter that appeared at that location), and then rated their subjective visibility for each of the two on a scale from *1* (not seen) to *4* (clearly seen). Critically, all responses were to be given in the order in which the targets had appeared, and irrespective of whether or not they had been consciously perceived. Participants were told to guess the locations of unseen squares. To enable the objective quantification of sensitivity to the presence of the masked targets, 20% of all trials served as a target-absent control condition: Either just the first target (partially target-present trial), just the second (partially target-present trial), or both target stimuli were replaced by the presentation of a blank screen.

### Visibility ratings accurately reflect subjective perception

We first evaluated our participants’ ability to detect the masked squares independently for each target. Subjective visibility ratings for both targets varied as a function of target presence. Target 1 was reported as seen on the majority of target-present trials (visibility > *1*; 80.4 ± 15.0% [*M* ± *SD*]), but was primarily rated as unseen on the target-absent catch trials (visibility = *1*; 76.6 ± 18.0%). Similarly, subjects indicated having seen a large proportion of target 2 when it was present (80.1 ± 16.5%), and judged most of the target-absent trials as unseen (81.5 ± 14.5%). Detection *d’* exceeded chance in both cases (target 1: 1.87 ± 0.80; *t*(37) = 14.35, *p* < 0.001; target 2: 2.04 ± 0.83; *t*(37) = 15.10, *p* < 0.001). Visibility reports were not fully independent, as the two targets tended to be either both perceived or both unperceived (Supplementary Fig. S1). Overall, then, participants used the visibility scale appropriately.

### Both targets can be maintained non-consciously

**Figure 2:**
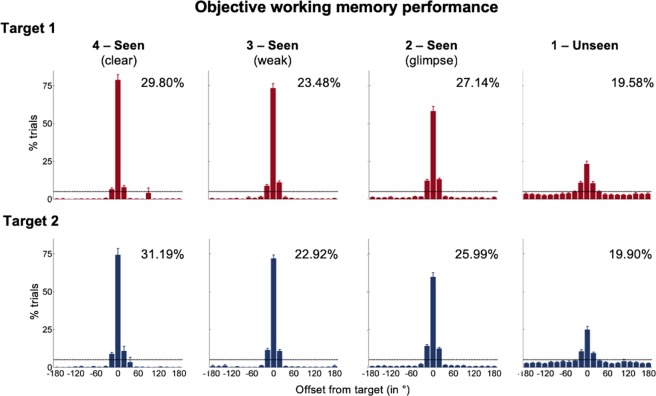
Objective performance for both targets. Spatial distributions of forced-choice localization performance in the working memory task are shown separately for target 1 (red) and target 2 (blue; 0 = correct target location; positive = counter-clockwise offset). Error bars indicate standard error of the mean (SEM) across subjects. The horizontal, dotted line illustrates chance level at 5%. Percentages show proportion of all available trials, on which the target under consideration had been presented (i.e., combining both fully present as well as partially present trials) and participants had reported the corresponding visibility.

As in our previous work^13^, subjects’ localization responses for both targets were centered on the correct position (Fig. 2). Accuracy was high on seen trials for target 1 (72.5 ± 15.4%) and increased monotonically with visibility, from rating *2* to rating *4* (pairwise comparisons: *t*s > 2.15, *p*s < 0.040). A similar pattern of findings also emerged for target 2. Overall accuracy for seen targets was comparable, though a bit lower, than the one for the first target (70.7% ± 16.2%; *t*(37) = 2.28, *p* = 0.028). It again varied as a function of visibility (pairwise comparisons: *t*s > 4.78, *p*s < 0.001, with the exception of the contrast between visibility *3* and *4*, where *t*(30) = 0.78 and *p* = 0.440). Crucially, even when participants had not seen the target in question (rating = *1*), they still identified the correct position much better than chance (chance = 5%; target 1: 23.2 ± 12.1%; *t*(37) = 9.25, *p* < 0.001, 95% CI = [14.2%, 22.1%]; Cohen’s d = 1.69; target 2: 24.9 ± 12.7%; *t*(37) = 9.68, *p* < 0.001, 95% CI = [15.8%, 24.1%]; Cohen’s d = 1.78). This long-lasting blindsight effect was of similar magnitude for both targets (*t*(37) = 1.06, *p* = 0.298, Bayes’ Factor = 0.29).

To gain more detailed insights into localization performance, we next summarized objective working memory performance with two complementary measures^13^: (1) The rate of correct responding quantified the amount of information that could be stored in working memory and was defined as the proportion of trials within ±2 positions (i.e., ±36°) of the actual target location. (2) The precision of subjects’ working memory representations was estimated as the standard deviation of that part of the original distribution reflecting genuine working memory (i.e., the spread within the zone of correct responding). It was only computed for those 32 of the 38 participants who exhibited above-chance blindsight for both target stimuli (i.e., chance = 25%; *p* < 0.050 in a χ^2^-test; Supplementary Fig. S2).

Subjects’ rates of correct responding largely mirrored the pattern of results obtained for accuracy. On seen trials, there was a trend towards a monotonic increase from visibility *2* to visibility *4* for target 1 (pairwise comparisons: *t*s > 1.83, *p*s < 0.077, with the exception of the contrast between visibility *3* and *4*, where *t*(30) = −0.44 and *p* = 0.664) and target 2 (pairwise comparisons: *t*s > 1.91, *p*s < 0.066). Importantly, even on unseen trials, participants’ rates of correct responding strongly exceeded chance for both targets (chance = 25%; target 1: 54.11 ± 17.23%; *t*(37) = 10.42, *p* < 0.001; target 2: 53.63 ± 15.41%; *t*(37) = 11.45, *p* < 0.001), although, in both instances, performance remained well below the level we had observed even for glimpsed seen targets (target 1 at visibility *2*: 86.57 ± 15.86%, *t*(37) = 13.73, *p* < 0.001; target *2* at visibility 2: 89.55 ± 14.24%; *t*(37) = 14.06, *p* < 0.001).

Working memory precision closely followed these findings. It again rose monotonically with increasing visibility for both targets (pairwise comparisons: *t*s > −2.17, *p*s < 0.040, with the exception of the comparisons between visibility *3* and *4*, where *t*(25) = −1.51, *p* = 0.143 for target 1, and *t*(25) = −0.10, *p* = 0.923 for target 2), and was reduced even in contrast to glimpsed seen targets on unseen trials (*t*s < −5.87, *p*s < 0.001).

Note that, intriguingly, those subjects with the highest accuracy and/or rate of correct responding on seen trials (collapsed across visibilities *2*–*4*) also tended to have the largest blindsight performances for both targets (accuracy: both Pearson’s *r*s > 0.30, both *p*s < 0.073; rates of correct responding: both Pearson’s *r*s > 0.42, both *p*s < 0.009). No such correlations were observed for participants’ precision for conscious and non-conscious working memory representations (both Pearson’s *r*s < 0.13, both *p*s > 0.489; Supplementary Fig. S3). A common third variable (e.g., attentional resources, intellectual capacities, etc.) may thus have influenced the amount of information that can be retained in both conscious and non-conscious working memory.

Overall then, we replicated and extended prior results^9,10,13^, demonstrating that both targets could be retained in non-conscious working memory over a long delay.

### Temporal order is maintained for seen and unseen targets

We next set out to evaluate objective performance for target 1 and target 2 as a function of joint visibilities. If indeed it were possible to retain information about temporal order in non-conscious working memory, location reports should remain accurate even if none of the targets had been seen. Furthermore, the size of these effects should be similar to when either only one target had been detected or only one had been presented.

Subjects’ ability to identify the correct target location depended on visibility, but not on temporal order. Overall, participants retained more and more precise information when they had seen rather than when they had not seen the targets (rate of correct responding: seen = 93.9 ± 9.2%, unseen = 53.9 ± 15.4%, *F*(1, 37) = 322.95, *p* < 0.001; precision: seen = 9.1 ± 2.4°, unseen = 16.3° ± 3.1°, *F*(1, 31) = 114.05, *p* < 0.001). Ordinal position, by contrast, did not affect localization reports and there were no significant interactions, suggesting that subjects had maintained the spatial position equally well for the two targets (rate of correct responding: target 1 = 73.8 ± 11.7%, target 2 = 74.0 ± 10.5%, *F*(1, 37) = 0.08, *p* = 0.780; precision: target 1 = 12.6 ± 2.3°, target 2 = 12.9 ± 2.4°, *F*(1, 31) = 0.40, *p* = 0.534; interaction effects: both *F*s < 0.78, both *p*s > 0.384).

**Figure 3:**
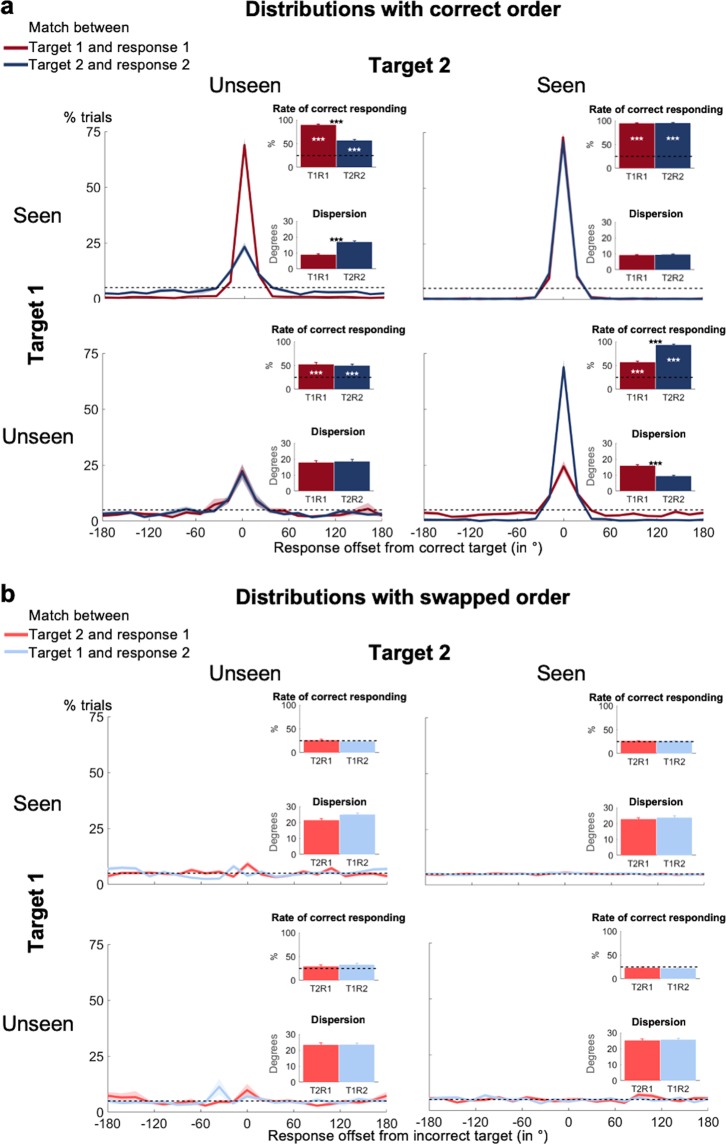
Temporal order can be maintained in non-conscious working memory. Spatial distributions of forced-choice localization performance in the working memory task on trials with two targets are shown as a function of visibility (i.e., seen vs. unseen) for target 1 and target 2 (0 = correct target location; positive = counter-clockwise offset). Distributions in the (**a**) upper panel reflect angular distances between target 1 and response 1 (T1R1) and target 2 and response 2 (T2R2), while distributions in the (**b**) lower panel reflect angular distances between target 1 and response 2 (T1R2, light blue) and target 2 and response 1 (T2R1, light red). Insets show rate of correct responding (within ±2 positions of actual location) and precision of working memory representations separately for seen and unseen trials. Error bars indicate standard error of the mean (SEM) across subjects. The horizontal, dotted line illustrates chance level at 5%. White asterisks show statistical significance when compared to chance (i.e., 25%) and black asterisks when comparing performance for target 1 with performance for target 2 (*p < 0.05, **p < 0.01, ***p < 0.001 in a paired-samples *t*-test, Bonferroni-corrected for 8 comparisons when comparing against chance, and for 4 comparisons when comparing performance for target 1 with performance for target 2).

Indeed, for the same visibility category (i.e., seen vs. unseen), the distributions of participants’ localization responses looked virtually identical, irrespective of whether they pertained to the first or the second target (Fig. 3a). Whenever subjects had detected both targets, they were near perfect in localizing both of them, with comparably high levels of correct responding and similar precision (Tables 1 and 2; Fig. 3a, top left). This performance for seen targets was remarkably stable for different pairings of visibility and target presence (Fig. 3a and Supplementary Fig. S4a). We observed no systematic effects for any one joined visibility condition to suggest different working memory performance in terms of storage or precision. Participants’ localization responses for seen targets were thus highly reproducible, irrespective of their visibility for the other target.

**Table 1:** Summary statistics for the rate of correct responding for target 1 and target 2 as a function of visibility.

Visibility	Target 1		Target 2		Paired-samples *t* test		
	*M*	*SD*	*M*	*SD*	*t*	*p*	BF
SeenSeen	94.2%	7.7%	94.9%	9.2%	−1.49	0.145	0.48
SeenUnseen	89.6%	13.4%	56.5%	18.4%	9.97	**<0.001**	1.33 * 10^9^
UnseenSeen	56.1%	19.4%	93.2%	13.3%	−11.02	**<0.001**	2.58 * 10^10^
UnseenUnseen	52.5%	24.9%	49.7%	21.0%	0.70	0.486	0.22
SeenAbsent	94.8%	13.7%	—	—	—	—	—
UnseenAbsent	51.1%	24.6%	—	—	—	—	—
AbsentSeen	—	—	93.9%	16.8%	—	—	—
AbsentUnseen	—	—	47.0%	32.4%	—	—	—

Note: Statistical comparison was carried out between targets. BF = Bayes’ Factor.

**Table 2:** Summary statistics for the precision for target 1 and target 2 as a function of visibility.

Visibility	Target 1		Target 2		Paired-samples *t* test		
	*M*	*SD*	*M*	*SD*	*t*	*p*	BF
SeenSeen	9.0°	2.7°	9.6°	2.4°	−2.59	**0.014**	3.25
SeenUnseen	8.9°	3.6°	16.9°	4.5°	−7.21	**<0.001**	344,125.89
UnseenSeen	15.8°	4.8°	9.2°	3.8°	5.54	**<0.001**	4,300.18
UnseenUnseen	17.7°	7.2°	18.5°	7.2°	−0.55	0.587	0.22
SeenAbsent	7.5°	3.6°	—	—	—	—	—
UnseenAbsent	20.0°	8.0°	—	—	—	—	—
AbsentSeen	—	—	8.9°	5.2°	—	—	—
AbsentUnseen	—	—	17.8°	10.1°	—	—	—

Note: Statistical comparison was carried out between targets. BF = Bayes’ Factor.

The picture was even clearer for unseen target squares. Subjective experience of the other target did not modulate objective localization performance at all. When only one of the targets had been seen (Fig. 3a) or when one of the targets had not been displayed (Supplementary Fig. S4a), subjects still responded correctly to the unseen target much more frequently than predicted by chance (chance = 25%; all rates of correct responding > 47.0%; all *p*s < 0.001, all Bayes’ Factors > 1.30 * 10^7^). The size and precision of this blindsight effect was comparable across all conditions (rate of correct responding: *t*s < 2.21, *p*s_corr_ > 0.510, Bayes’ Factors < 1.56; precision: *t*s < 2.82, *p*s_corr_ > 0.135, Bayes’ Factors < 5.12).

Crucially, even when participants had missed both targets, they still exhibited long-lasting blindsight for both of them (rates of correct responding >49.7%; *p*s < 0.001, Bayes’ Factors > 1.29 * 10^13^; Fig. 3a, bottom left). This effect was equally strong and precise for target 1 and target 2 (rate of correct responding: 52.5% vs. 49.7%; *t*(36) = 0.70, *p* = 0.486, Bayes’ Factor = 0.22; precision: 17.7° vs. 18.5°; *t*(30) = −0.55, *p* = 0.587, Bayes’ Factor = 0.22), and did not differ from the performance in any of the other conditions (rate of correct responding: *t*s < 2.01, *p*s_corr_ > 0.052, Bayes’ Factors < 1.08; precision: *t*s < 2.03, *p*s_corr_ > 0.052, Bayes’ Factors < 1.15). Just as was the case for conscious working memory, the non-conscious maintenance of a target stimulus was therefore unaffected by subjects’ visibility of the other target. Moreover, on trials in which none of the target squares had been seen, the characteristics of the blindsight effect were highly similar for target 1 and target 2, suggesting that participants could non-consciously retain two targets in correct temporal order. To fully support this conclusion, however, several controls are needed.

### No evidence for swapping errors for seen and unseen targets

The results so far remain compatible with the possibility that subjects maintained the order of unseen targets on a subset of trials, but reported them in incorrect order on other trials. In our paradigm, this would translate into swapping errors, with participants choosing the location of the second target for their first response and vice versa.

To investigate this possibility, we examined the response distributions assuming that subjects had performed swapping errors; that is, we calculated the distance between target 1 and response 2, and between target 2 and response 1 as a function of visibility for both targets. The distributions for both seen and unseen targets were almost entirely flat (Fig. 3b), indicating the absence of swapping errors. Whenever participants had detected at least one of the targets, there was no discernable above-chance performance (all rates of correct responding <26.4%, all *p*s_corr_ > 0.120, all Bayes’ Factors < 3.31) or differences between conditions in terms of rate of correct responding or precision (rate of correct responding: *t*s < 2.98, *p*s_corr_ > 0.075, Bayes’ Factors < 7.47; precision: *t*s < 2.78, *p*s_corr_ > 0.135, Bayes’ Factors < 4.71). This pattern persisted when one of the targets had been absent (Tables 3 and 4; Supplementary Fig. S4b).

**Table 3:** Summary statistics for the rate of swapping errors for target 1 and target 2 as a function of visibility.

Visibility	Target 1 – Response 2		Target 2 – Response 1		Paired-samples *t* test		
	*M*	*SD*	*M*	*SD*	*t*	*p*	BF
SeenSeen	26.4%	3.7%	26.1%	3.6%	−0.75	0.456	0.23
SeenUnseen	23.6%	12.4%	25.9%	15.1%	1.16	0.253	0.28
UnseenSeen	22.0%	8.5%	23.1%	12.2%	0.47	0.644	0.19
UnseenUnseen	32.5%	20.1%	29.4%	19.1%	−0.84	0.408	0.25
SeenAbsent	23.5%	17.7%	—	—	—	—	—
UnseenAbsent	32.5%	21.9%	—	—	—	—	—
AbsentSeen	—	—	27.5%	22.1%	—	—	—
AbsentUnseen	—	—	28.9%	28.1%	—	—	—

Note: Statistical comparison was carried out between targets. While *p*-values reported in the text have been corrected for multiple comparisons, *p*-values reported in this table reflect values prior to correction. BF = Bayes’ Factor.

**Table 4:** Summary statistics for the precision related to the swapping errors for target 1 and target 2 as a function of visibility.

Visibility	Target 1 – Response 2		Target 2 – Response 1		Paired-samples *t* test		
	*M*	*SD*	*M*	*SD*	*t*	*p*	BF
SeenSeen	23.6°	7.6°	22.6°	5.9°	−0.52	0.608	0.22
SeenUnseen	24.8°	5.7°	21.3°	6.9°	−2.78	**0.009**	4.71
UnseenSeen	25.6°	5.2°	25.2°	5.5°	−0.43	0.670	0.21
UnseenUnseen	23.5°	5.9°	23.3°	7.6°	−0.28	0.781	0.21
SeenAbsent	23.1°	6.0°	—	—	—	—	—
UnseenAbsent	22.7°	7.6°	—	—	—	—	—
AbsentSeen	—	—	25.6°	5.9°	—	—	—
AbsentUnseen	—	—	22.3°	7.6°	—	—	—

Note: Statistical comparison was carried out between targets. While *p*-values reported in the text have been corrected for multiple comparisons, *p*-values reported in this table reflect values prior to correction. BF = Bayes’ Factor.

Crucially, for the critical case of two unseen targets, we also obtained similar findings (Fig. 3b, bottom left). Note that the small peaks one seems to notice within the region of correct responding for both distributions did not cross the threshold for statistical significance (rates of correct responding < 32.5%, *p*s_corr_ > 0.120, Bayes’ Factors < 3.27) and, importantly, almost disappeared entirely when we excluded all trials, in which target 1 and target 2 had the same position (rates of correct responding <30.4%, *p*s > 0.056, Bayes’ Factors < 1.10). The “rate of correct responding” and “precision” were also significantly better when classified based on true than on swapped order (rate of correct responding: all *t*s > 4.12, all *p*s_corr_ < 0.001, all Bayes’ Factors > 125.39; precision: all *t*s > 3.12, all *p*s_corr_ < 0.004, all Bayes’ Factors > 9.52).

When directly contrasting distributions with the correct versus incorrect temporal order in a single repeated-measures ANOVA, we observed the expected visibility (i.e., seen vs. unseen) by type of distribution (i.e., correct vs. incorrect temporal order) interactions (rate of correct responding: *F*(1, 37) = 344.86, *p* < 0.001; precision: *F*(1, 31) = 37.49, *p* < 0.001). While visibility modulated responses based on true order, it did not affect the distributions based on swapped order. In summary, there was no evidence for temporal-order swapping errors, even on purely unseen trials when subjects reported not having seen any of the targets.

### Long-lasting blindsight effects for both targets can occur on the same trial

We have established that (1) a comparable blindsight effect exists for target 1 and target 2, even when neither had been seen (Fig. 3a), and that (2) the distributions reflecting genuine working memory of ordered information serve as a better predictor of behavior than do the swapped distributions (Fig. 3b). Nevertheless, it could still be the case that participants accurately reported target 1 on some trials, and target 2 on other trials, but not both on the same trial.

**Figure 4:**
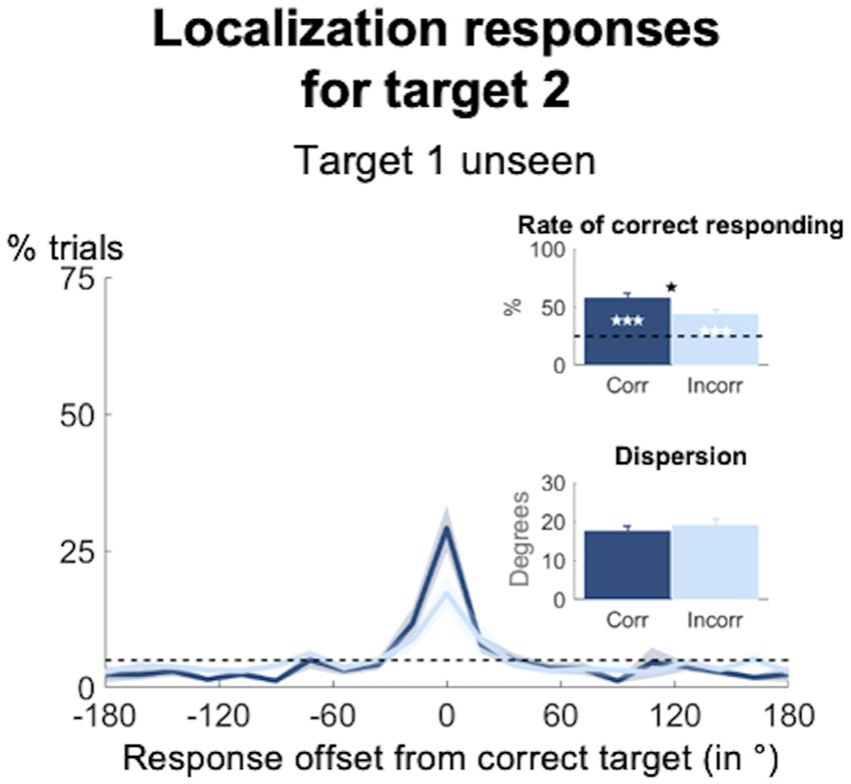
Long-lasting blindsight effect may occur simultaneously. Spatial distributions of forced-choice localization performance for unseen target 2 as a function of whether the position for unseen target 1 had been identified correctly (dark blue) or not (light blue; 0 = correct target location; positive = counter-clockwise offset). Distributions reflect angular distances between target 2 and response 2. Insets show rate of correct responding (within ±2 positions of actual location) and precision of working memory representations separately for trials on which target 1 had been unseen correct and trials on which it had been unseen incorrect. Error bars indicate standard error of the mean (SEM) across subjects. The horizontal, dotted line illustrates chance level at 5%. White asterisks show statistical significance when compared to chance (i.e., 25%) and black asterisks when comparing performance as a function of accuracy for target 1 (*p < 0.05, **p < 0.01, ***p < 0.001 in a paired-samples *t*-test).

As can be seen in Fig. 4, this did not turn out to be so. When restricting our trials to just that subset, on which target 1 had not been seen, yet localized correctly, we still observed above-chance performance for the second target (rate of correct responding: 57.8 ± 26.4%; *t*(35) = 7.46, *p* < 0.001, Bayes’ Factor = 2.59*10^6^). A slightly less pronounced blindsight effect also emerged for target 2 following incorrectly localized first targets (rate of correct responding: 44.0 ± 23.7%; *t*(35) = 4.83, *p* < 0.001, Bayes’ Factor = 1653.02; paired-samples *t*-test: *t*(34) = 2.61, *p* = 0.013, Bayes’ Factor = 3.32). Both effects were comparable to the non-conscious working memory performance we had observed for target 1 on trials on which neither target had been detected (all *t*s < 1.65, all *p*s_corr_ > 0.108, all Bayes’ Factors < 0.61) or on which only the first had been presented (all *t*s < 1.06, all *p*s_corr_ > 0.298, Bayes’ Factors < 0.30). They also did not differ from the rate of correct responding for target 2, when the first target had been omitted (all *t*s < 1.37, all *p*s_corr_ > 0.181, Bayes’ Factors < 0.43). As such, there indeed existed a subset of trials on which two targets were simultaneously maintained non-consciously in proper order.

## Discussion

Previous research on non-conscious working memory has almost exclusively focused on the maintenance of single, sensory items^8–10,13^. We here replicated and critically extended this earlier work: Subjects were able to not only store two unseen targets simultaneously, but also to retain their temporal order.

Consistent with prior findings, we observed a long-lasting blindsight effect for both targets. Even when participants had reported not having seen the target stimulus, they identified its location much better than predicted by chance – up to ~4 s after its presentation. The magnitude and precision of this above-chance objective performance in the absence of subjective awareness remained constant throughout the entire experiment: It neither varied as a function of the number of targets presented (i.e., one vs. two), nor as a function of the ordinal position of the target (i.e., first vs. second). Crucially, it also persisted when neither of the two targets had been detected and, importantly, still occurred for the second target when the first target had been localized correctly. Subjects were thus clearly able to simultaneously retain two target locations in non-conscious working memory, at least on a subset of trials. They also committed virtually no swapping errors, almost exclusively reporting the location of the two targets in the proper order. In addition to maintaining the identity of multiple items, participants therefore also stored their temporal order. Taken together, these results suggest that the competencies of non-conscious working memory may reach much further than previously shown and, within the realm of features addressed in the present experiment, share important commonalities with conscious working memory.

Which mechanism might have permitted to non-consciously maintain multiple items in addition to their temporal order? A first possibility is that subjects did not rely on non-conscious working memory at all, instead either accidentally miscategorizing some seen targets as unseen, or guessing the target positions immediately after their presentation and then consciously maintaining their ordered identity. In the context of the present experiment, we cannot fully reject this possibility. However, we deem it unlikely for several reasons. First, we carefully instructed participants on the appropriate use of the visibility rating scale, stressing that a rating of *1* should be reserved exclusively for those trials, on which they thought the target to be absent. Second, having combined an almost identical paradigm with magnetoencephalography recordings, we have previously shown that non-conscious maintenance is genuine^13,20^: Subjects neither erroneously miscategorized their visibility ratings nor consciously maintained an early guess. Indeed, when comparing the size of the blindsight effect (i.e., rate of correct responding) obtained in our first study with the ones obtained for target 1 and target 2 in the present experiment, we found no evidence for any differences (independent samples *t*-test: both *t*s < 1.64, both *p*s > 0.108, both Bayes’ Factors < 0.85). Note that, based on an analysis of reaction time data, other groups have similarly argued in favor of genuine non-conscious working memory^9^. Taken together, this evidence supports the hypothesis of a long-lasting blindsight effect in the present experiment.

On the theoretical level, the non-conscious maintenance of multiple representations with temporal order is also fully compatible with the main brain mechanism proposed for non-conscious working memory. An emerging view is that, in contrast to conscious working memory, contents in non-conscious working memory may be maintained in activity-silent brain states^13,21,22^. According to this framework, temporary shifts in synaptic weights may effectively link populations of neurons, thereby allowing networks to go silent, while still leaving behind a transient synaptic memory trace of their previously active configuration for several seconds^14,22^. This original computational model of synaptic working memory already included simulations of the storage of multiple items^14,23^. The key idea is straightforward: Individual memories are retained by item-specific patterns of synaptic facilitation. If more than a single item is to be stored, the neuronal networks coding for the individual contents reactivate consecutively in brief bursts of activity separated by long activity-silent periods, thereby enabling the short-term maintenance of several representations. Moreover, recent models of serial order representations in working memory assume that prefrontal neurons may code conjunctively for item and order: The neural code for item *i* at temporal position *t* would be a vector resulting from the tensor product of vectors coding for *i* and for *t*^24^. Assuming such a neural code, any non-specific signal, such as our recall cue, would reactivate the population coding for a specific item, thereby allowing downstream systems to read out the stored information. If the stored pattern itself contains information about item identity as well as temporal order, retrieving one piece of information would imply retrieving the other. The combination of the Botvinick *et al*.^24^ and the Mongillo *et al*.^14^ mechanisms therefore provides a straightforward mechanism for the storage of multiple representations and their temporal order in activity-silent brain states. Future research might directly test this proposal at the brain level.

Our work raises the issue of the capacity limits of conscious and non-conscious working memory. Perhaps the most defining characteristic of working memory is its capacity-limited nature. In stark contrast to other forms of short-term memory, such as iconic^25^ or fragile memory^26^, only about ~4 to 7 items may concurrently be stored in working memory^27^. In the current experiment, we demonstrated that both the amount of information as well as the precision with which this information could be retained in non-conscious working memory was largely unaffected by the number of items originally encoded into non-conscious working memory. That is, the long-lasting blindsight effect remained the same, irrespective of whether participants missed the only target present (on partial target-absent trials), failed to detect one of the two targets, or did not see either one. However, we only tested memory for two items, which appears to fall well within the capacity limits of conscious working memory. In the future, it will be important to directly evaluate the capacity of non-conscious working memory. In light of the proposed activity-silent mechanism underlying maintenance in non-conscious working memory^13^, we speculate that, if any such limits do exist, they might be more closely related to the number and/or quality of the memory traces laid down during encoding or accessed during retrieval than to the quantity and/or precision of the stored representations themselves (as is assumed to be the case for conscious working memory).

## Conclusion

Recently, there has been growing interest and evidence for the notion of a genuine non-conscious working memory. However, the precise nature of this phenomenon is still unclear. Our work critically expands our understanding of this long-lasting blindsight effect. Combining a masking paradigm with a spatial delayed-response task, we demonstrated that non-conscious working memory may accommodate the storage of multiple items as well as their temporal order. We further propose that these capacities arise from activity-silent mechanisms, believed to support maintenance in non-conscious working memory. As such, our results highlight the similarities between conscious and non-conscious working memory and continue to challenge current conceptualizations of working memory based on conscious processing and sustained neural activity.

## Methods

### Subjects

All experimental procedures had been approved by the Ethics Committee on Human Research at Neurospin (Gif-sur-Yvette, France) and were carried out in accordance with the guidelines of the Declaration of Helsinki. Based on our previous observation of a large effect size for non-conscious working memory performance^13^ and a desired statistical power of 0.90 at an α-level of 0.050, we recruited a total of 40 healthy volunteers (24 women; *M*_age_ = 24.85 years, *SD*_age_ = 4.20 years). All subjects had normal or corrected-to-normal vision, presented themselves without a history of neurological or psychiatric antecedents, and gave written informed consent prior to participation. They received €20 as compensation for their time and effort. Due to non-compliance with task instructions, we excluded 2 participants, resulting in a final dataset of 38 subjects.

### Working memory task

Participants performed a variant of our masked, spatial delayed-response protocol^13^ to evaluate the short-term maintenance of sequences of memoranda as well as abstract order information in conscious and non-conscious working memory (Fig. 1). The experiment was programmed and presented using Psychtoolbox software (http://psychtoolbox.org/), run in a Matlab R2017 environment. Each trial began with a 1 s fixation period, followed by the presentation of the first target stimulus: A faint, gray square was briefly displayed in 1 out of 20 circular locations (17 ms). After a short inter-stimulus interval (ISI) of 17 ms, a visual mask, whose contrast had been calibrated on an individual basis to produce roughly equal proportions of seen and unseen targets (see below), appeared in all possible positions (233 ms), effectively camouflaging the target location. This sequence of events (i.e., target, ISI, mask) was then repeated a second time, separated from the initial one by an 833 ms delay. Importantly, we drew locations for both targets independently of each other (such that, on a small subset of trials, successive targets could appear in the same position), and ensured a fully counterbalanced design, with all possible dependencies between target 1 and target 2 occurring with equal probability. Target-absent catch trials, on which the presentation of the target square was replaced by a blank screen, were also included to allow for an objective quantification of our subjects’ sensitivity to the target stimuli: While 4% of all trials contained no target square at all (target-absent trials), an additional 16% omitted either just the first or the second target (partially target-present trials).

A given trial then terminated with two successive responses: Participants first identified the spatial locations of the two targets in the order they had appeared, and then rated their subjective visibility of both target squares on the 4-point Perceptual Awareness Scale^28^. Both types of responses were entered on a standard AZERTY keyboard. On each trial, a subset of lower-case letters of the alphabet (excluded: *b*, *c*, *j*, *n*, *p*, *t*) was randomly placed in the 20 positions, permitting subjects to simply type the letter corresponding to the location in question. The number pad keys were used to indicate visibility. Crucially, target localization was required on all trials and under all circumstances. Even when participants had not seen a given target square, we instructed them to choose a position, guessing it if necessary. Moreover, subjects were to only declare a target as unseen (i.e., visibility = *1*), if they had not perceived it at all; in case of the slightest doubt, they had to rate it as seen (i.e., visibility > *1*). The inter-trial interval (ITI) was jittered between 333 ms and 666 ms. Background color of the screen was set to black (RGB: 1, 1, 1), and all other stimuli, with the exception of the target and mask, were shown in white (RGB: 255, 255, 255). We constantly presented a central fixation cross in order to aid participants in orienting their gaze and attention onto the center of the screen throughout the entire experiment. Overall, subjects completed 500 trials of this task, split into 10 blocks of 50 trials each, and presented on a flat screen computer monitor (viewing distance ~60 cm) in a dimly lit testing cabinet.

### Calibration task

Just before the main experimental task, participants also completed 100 trials of a separate calibration procedure, designed to estimate the mask contrast needed for roughly equal proportions of seen and unseen targets. Up until (and including) the presentation of the first mask, this calibration was strictly identical to the main working memory paradigm. It then, however, diverged, requiring an immediate rating of subjective visibility without the need to maintain multiple targets or the order of their presentation. Crucially, we applied a double-staircase technique to adjust mask contrast at the single-trial level. Whenever subjects had rated a target-present trial as unseen (visibility = *1*), mask contrast was reduced by 1/20^th^ on the subsequent trial. By contrast, it was increased by the same amount whenever participants had reported a target-present trial as seen (visibility >*1*). Initial values for the two staircases were set to RGB values of 12.75, 12.75, 12.75 and 242.5, 242.5, 242.5, respectively, and one of the two staircases was randomly selected at the beginning of each trial. In case of target-absent trials, the previous mask contrast from a randomly chosen staircase was re-used without having been updated. We then computed individual mask contrasts to be used in the main task by taking the grand average over the last four switches (i.e., from seen to unseen or vice versa) across the two staircases. The same contrast was applied for the two targets.

### Data analyses and statistics

In analogy to our previous approach^13^, we summarized objective working memory performance with three complementary measures. For each subject, target, and condition of interest, we computed (1) the accuracy, (2) the rate of correct responding, and (3) the precision of forced-choice localization responses. Whereas the former two both capture the quantity of information that may be retained, the latter serves as an estimate of the quality of the underlying working memory representations. Details on how exactly to derive all of these indices have already been provided in our previous open-access publication^13^, so we will only focus on the main elements here.

Accuracy simply corresponds to that proportion of trials for which participants had identified the exact target location, leading to a chance level of 5% (i.e., 1/20). The rate of correct responding, in contrast, takes into account small errors in localization performance, having been defined as that proportion of trials within close spatial proximity (i.e., ±2 positions) of the actual target location. Chance for this measure is 25% (i.e., 5/20). For participants displaying sufficient blindsight for both targets (i.e., *p* < 0.05 in a *χ*^2^-test against chance, collapsed across all other conditions), we also estimated the precision of that part of the distribution within the zone of correct responding corresponding to genuine working memory, after having accounted for random guessing (see^13^ for all details).

We submitted all of these indices to appropriate statistical tests, being either (1) one-sample *t*-tests (for comparisons against chance), (2) paired samples *t*-tests (for all comparisons requiring identification of which specific conditions might have differed), or (3) repeated-measures ANOVAs (for comparisons aiming at identifying just any overall effect). The statistical threshold for significance was set to *p* < 0.050, and, in the case of multiple comparisons, a Bonferroni correction was applied. In addition, where appropriate, we also provide Bayes’ Factors based on one- or two-sided *t*-tests (*r* = 0.707)^29^.

## Supplementary information

Supplementary Material

## Data Availability

The data from the current study are available from the lead author at darinkat87@gmail.com.
