# Supplementary material for: Temporal-order information can be maintained in non-conscious working memory
Source: Sci Rep. 2019 Apr 24;9:6484. doi: 10.1038/s41598-019-42942-z (PMC6482300; doi:10.1038/s41598-019-42942-z)
Supplement: Supplementary file 1 — Supplementary Material [file 41598_2019_42942_MOESM1_ESM.pdf]

Supplementary material for the paper

"Temporal-order information can be maintained in non-conscious working memory"

Darinka Trübutschek, Sébastien Marti, and Stanislas Dehaene

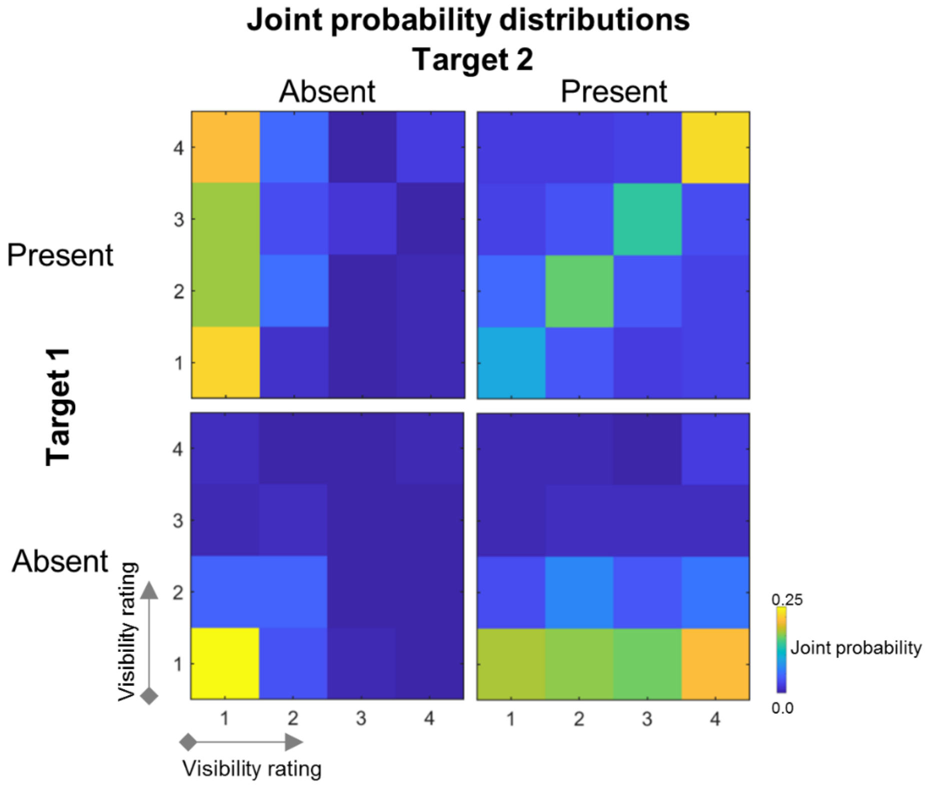

Supplementary Figure S1.
Partial dependence of visibility reports.
Joint probability distributions for all combinations of visibility for target 1 (vertical axis) and target 2 (horizontal axis) as a function of target presence and absence. Hotter colors reflect higher probability.

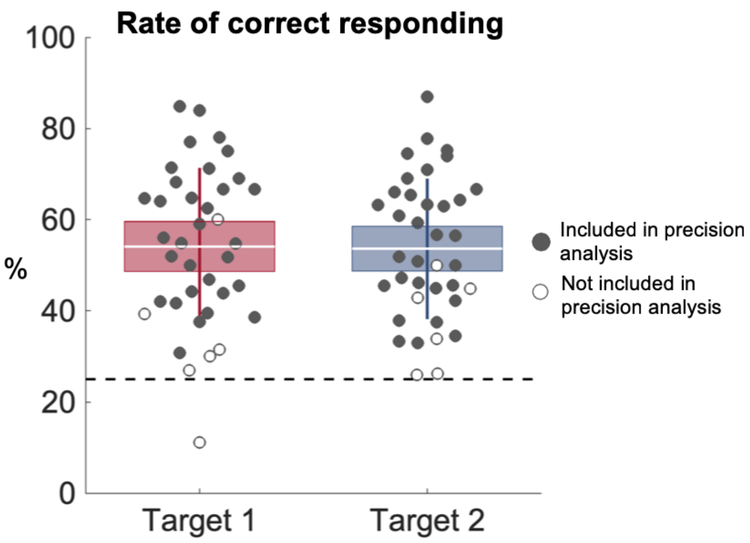

Supplementary Figure S2.
Individual blindsight performances.
Rates of correct responding are shown for individual subjects (circles) separately for target 1 (left) and target 2 (right). Filled circles reflect participants with sufficient blindsight to be included in the precision analysis (i.e., $p < 0.050$ in a χ^2-test against chance at 25%), open circles subjects that were excluded from this particular set of analyses. Horizontal white lines show the mean, colored vertical lines the respective standard deviations. Colored patches correspond to the 95% confidence intervals for the means. Dashed horizontal line indicates chance level at 25%.

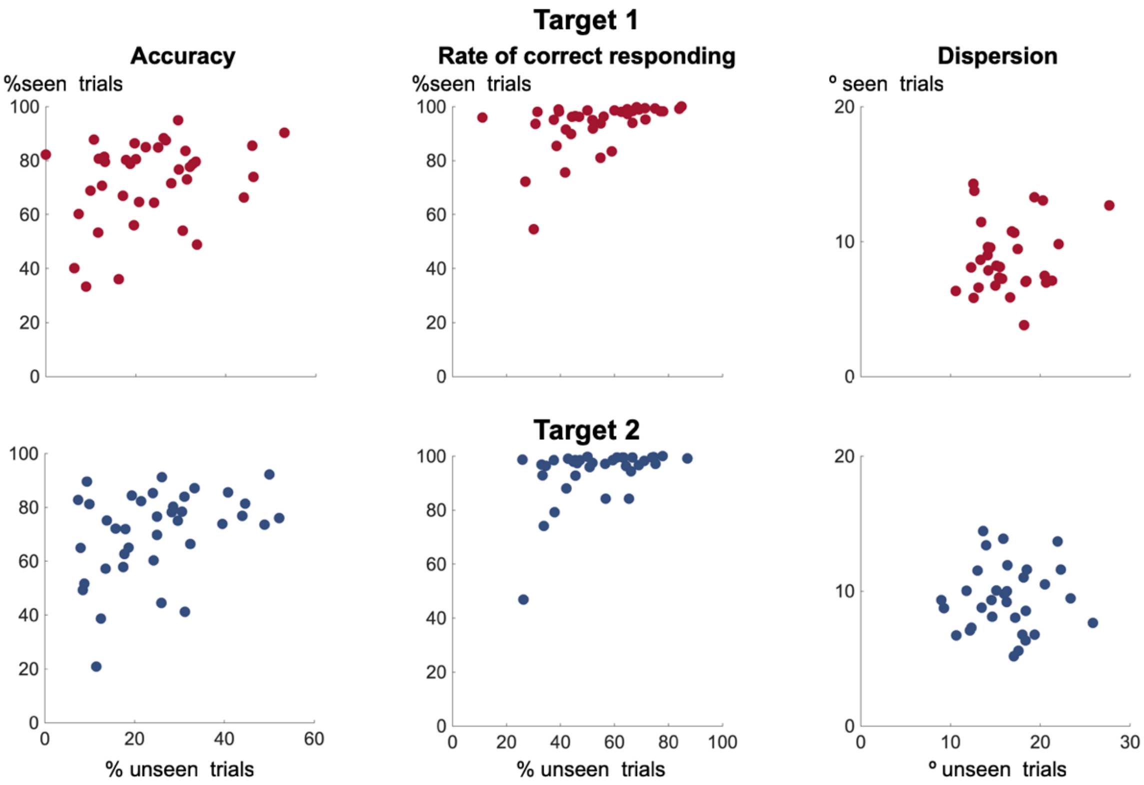

Supplementary Figure S3.
Amount of information retained in conscious working memory correlates with blindsight performance.
Scatter plots depicting the relationship between seen and unseen targets in terms of accuracy (left column), the rate of correct responding (middle column), and precision (right column) separately for target 1 (upper row) and target 2 (bottom row).

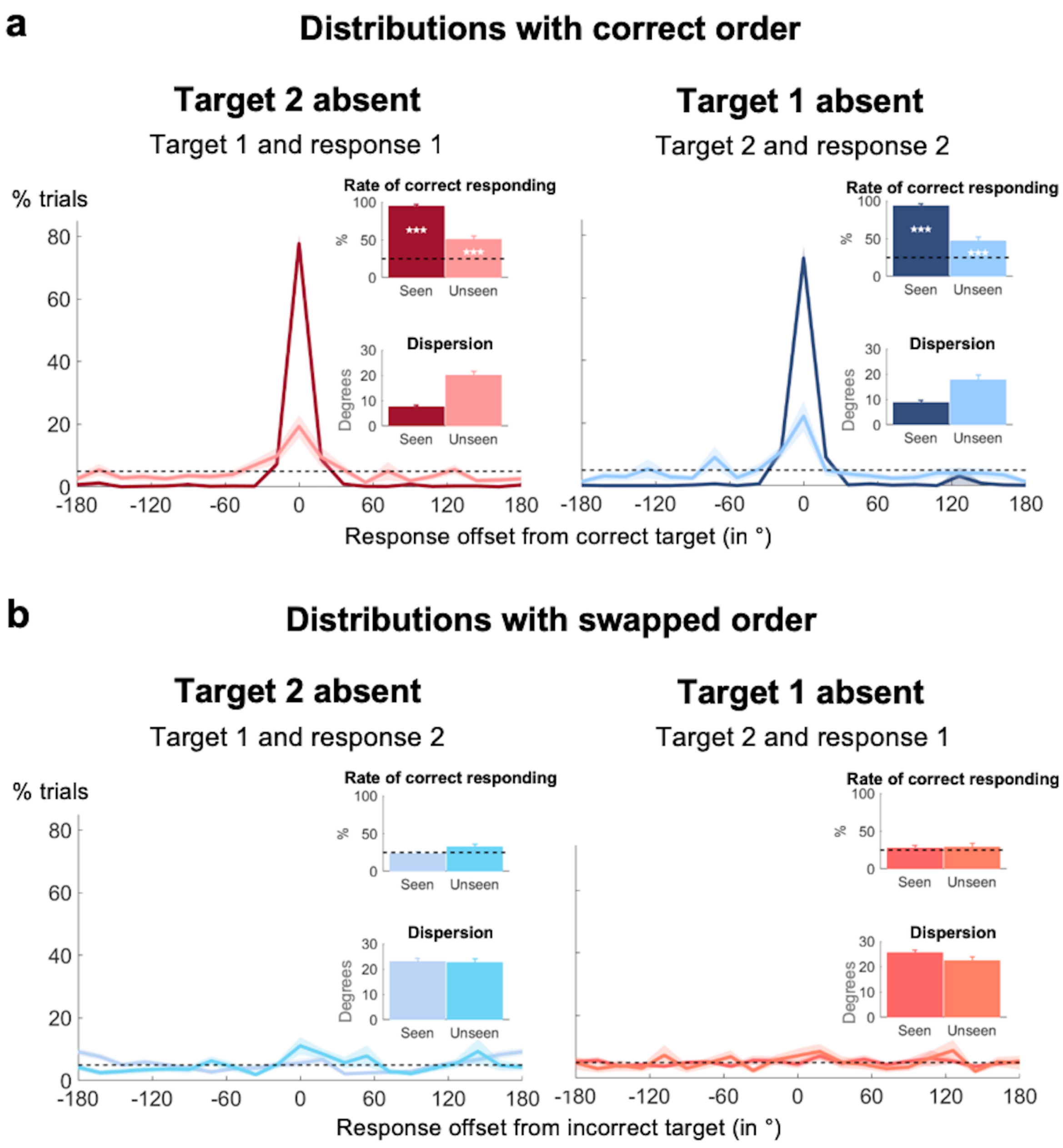

Supplementary Figure S4.

Target absence does not influence localization reports for the other target.

Spatial distributions of forced-choice localization performance in the working memory task on trials with one target are shown as a function of visibility (i.e., seen vs. unseen) for target 1 and target 2 (0 = correct target location; positive = counter-clockwise offset). Distributions in the **(a)** upper panel reflect angular distances between target 1 and response 1 and target 2 and response 2, while those in the **(b)** lower panel reflect angular distances between target 1 and response 2 and target 2 and response 1. Insets show rate of correct responding (within ±2 positions of actual location) and precision of working memory representations separately for seen and unseen trials. Error bars indicate standard error of the mean (SEM) across subjects. The horizontal, dotted line illustrates chance level at 5%. White asterisks show statistical significance when compared to chance (i.e., 25%; $*p < 0.05$, $**p < 0.01$, $***p < 0.001$ in a paired-samples t-test, Bonferroni-corrected for 4 comparisons).
